# An AI-based novel system for predicting respiratory support in COVID-19 patients through CT imaging analysis

**DOI:** 10.1038/s41598-023-51053-9

**Published:** 2024-01-08

**Authors:** Ibrahim Shawky Farahat, Ahmed Sharafeldeen, Mohammed Ghazal, Norah Saleh Alghamdi, Ali Mahmoud, James Connelly, Eric van Bogaert, Huma Zia, Tania Tahtouh, Waleed Aladrousy, Ahmed Elsaid Tolba, Samir Elmougy, Ayman El-Baz

**Affiliations:** 1 Department of Computer Science, Faculty of Computers and Information, Mansoura University, Mansoura, Egypt; 2 Department of Bioengineering, University of Louisville, Louisville, USA; 3 Electrical, Computer and Biomedical Engineering Department, Abu Dhabi University, Abu Dhabi, UAE; 4 Department of Computer Sciences, College of Computer and Information Sciences, Princess Nourah Bint Abdulrahman University, Riyadh, Saudi Arabia; 5 Department of Radiology, University of Louisville, Louisville, USA; 6 College of Health Sciences, Abu Dhabi University, Abu Dhabi, UAE; 7 The Higher Institute of Engineering and Automotive Technology and Energy, Kafr El Sheikh, Egypt

**Keywords:** Diagnosis, Translational research

## Abstract

The proposed AI-based diagnostic system aims to predict the respiratory support required for COVID-19 patients by analyzing the correlation between COVID-19 lesions and the level of respiratory support provided to the patients. Computed tomography (CT) imaging will be used to analyze the three levels of respiratory support received by the patient: Level 0 (minimum support), Level 1 (non-invasive support such as soft oxygen), and Level 2 (invasive support such as mechanical ventilation). The system will begin by segmenting the COVID-19 lesions from the CT images and creating an appearance model for each lesion using a 2D, rotation-invariant, Markov–Gibbs random field (MGRF) model. Three MGRF-based models will be created, one for each level of respiratory support. This suggests that the system will be able to differentiate between different levels of severity in COVID-19 patients. The system will decide for each patient using a neural network-based fusion system, which combines the estimates of the Gibbs energy from the three MGRF-based models. The proposed system were assessed using 307 COVID-19-infected patients, achieving an accuracy of $97.72\%\pm 1.57$, a sensitivity of $97.76\%\pm 4.08$, and a specificity of $98.87\%\pm 2.09$, indicating a high level of prediction accuracy.

## Introduction

**Figure 1:**
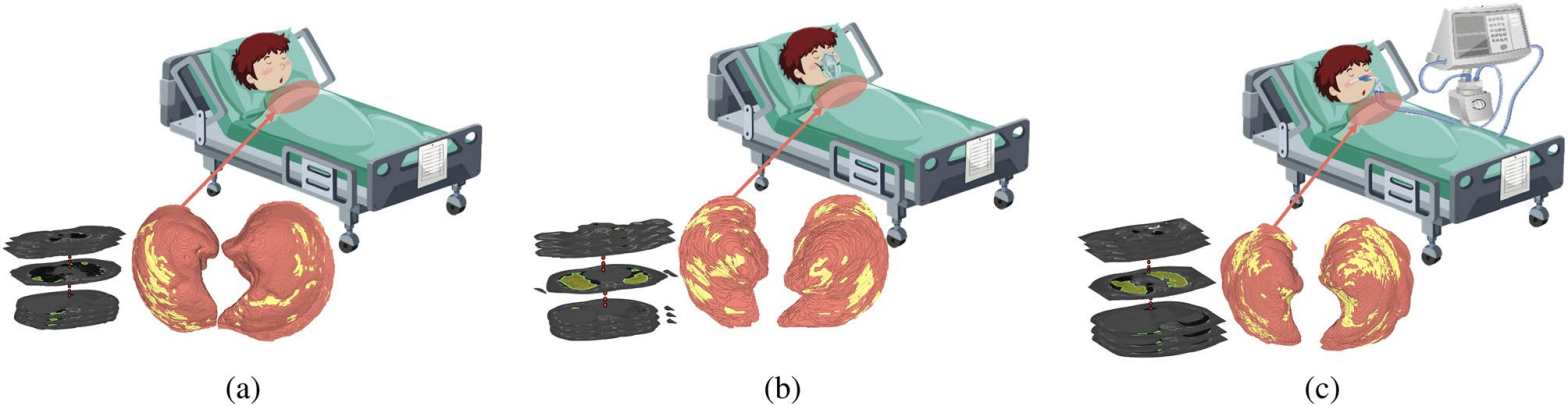
An illustrative example of the Correlation between CT lesions and respiratory support levels: (**a**) minimal support, (**b**) non-invasive support, and (**c**) invasive support.

The severe acute respiratory syndrome coronavirus 2 (SARS-CoV-2) virus emerged at the end of 2019 and caused the coronavirus disease of 2019 (COVID-19)^1^. The virus quickly spread across the world, resulting in severe impacts on the global economy and public health^2^. In March 2020, the World Health Organization (WHO) declared COVID-19 a global pandemic due to its rapid spread^3^. The severity of COVID-19 infection varied among individuals^4^. Based on the respiratory support provided to COVID-19 patients, the severity of COVID-19 infection was classified into three categories: minimal support (level 0), non-invasive support (level 1), and invasive support (level 2)^5^. Individuals with level-0 COVID-19 required minimal support for treatment, while those with level-1 COVID-19 had moderate symptoms and required non-invasive ventilation (e.g., soft oxygen) to recover. Individuals with level-2 COVID-19 had severe symptoms and required invasive ventilation (e.g., mechanical ventilation) for recovery. Figure 1 depicts the three respiratory support levels that COVID-19 patients may require during treatment and their correlation with CT images. COVID-19 has affected a staggering 758 million people worldwide, with 6.859 million deaths attributed to incorrect or delayed identification of COVID-19 severity^6^. Thus, identifying the severity of COVID-19 is crucial to determine the appropriate treatment for infected individuals^7^. Since the beginning of the pandemic, physicians have relied on imaging data such as X-ray and CT scans to diagnose the severity of COVID-19^8^. However, this method of classification has resulted in incorrect identification of severity, leading to inappropriate treatment and, in some cases, fatalities.

Many recent studies have proposed AI-based computer-aided diagnosis (CAD) systems that can detect COVID-19 severity, but they rely only on imaging data, leading to inaccurate results. In this paper, we present a new CAD system that predicts the respiratory support level (i.e., the severity of COVID-19) needed for each COVID-19 patient by analyzing the relationship between their CT scan volume and respiratory support, with the following contributions: (1) we propose an automatic segmentation system that delineates the lung regions from CT scans. (2) This proposed segmentation system is also used to extract lesion regions from region of interest selected by a radiologist. (3) A 3D rotation-invariant MGRF model is employed to represent the discrimination between different respiratory support levels. (4) Two stages of neural networks are utilized to predict the required respiratory support level for each patient by combining the results of each model.

## Related work

The emergence of COVID-19 led to the development of CAD systems for diagnosing and classifying its severity. Cabitza et al.^9^ developed machine learning models that accurately detect COVID-19 using blood test results. The models were built and validated with data from 371 COVID-19-positive patients and 526 COVID-19-negative patients, achieving an overall accuracy of 95.7%. Yao et al.^10^ developed a machine-learning model that predicts the severity of COVID-19 using routine blood and urine test results. They used data from 205 COVID-19 patients and employed feature selection techniques and various machine-learning algorithms to build and validate their model. The results revealed that the model accurately predicted the severity of COVID-19 based on the patient’s blood and urine test results. Brinati et al.^11^ investigated the feasibility of detecting COVID-19 infection from routine blood tests using machine learning algorithms. They collected data from 279 COVID-19-positive and 277 COVID-19-negative patients and utilized five different machine learning models to classify patients as COVID-19 positive or negative based on their blood test results. The results indicated that the models accurately detected COVID-19 infection from routine blood tests with an overall accuracy ranging from 83.3 to 97.8%. Aktar et al.^12^ proposed a CAD system for identifying COVID-19 disease using a Reverse Transcription-Polymerase Chain Reaction (RT-PCR) test. Their system had two phases: feature selection and diagnosis. The authors used chi-square, Pearson correlation, and Student t algorithms in the feature selection phase to choose blood parameters that could distinguish between healthy and COVID-19 patients. In the diagnosis phase, they employed different machine-learning algorithms, including a decision tree, random forest, gradient boosting machine, and support vector machine (SVM), to detect COVID-19 infection. The authors created a new dataset by combining two datasets to evaluate the system’s performance. The experimental results demonstrated that the random forest algorithm outperformed others with 92% accuracy. Zhang et al.^13^ proposed a CAD system that can determine the severity of COVID-19 infection by analyzing patients’ blood test results. The system categorizes patients into either mild or severe cases. The authors employed six different classifiers, including random forest, naive Bayes, SVM, k-nearest neighbors (KNN), logistic regression, and neural networks, to determine the severity of the disease. They tested the performance of the system on a dataset of 422 COVID-19-positive patients from the Shenzhen Third People’s Hospital. The dataset included 38 blood test features for each patient. The authors found that naive Bayes outperformed the other classifiers, with an area under the curve (AUC) of 0.90. Previous CAD systems had shown that classification based on blood tests alone could not achieve high accuracy, and researchers had started using imaging data, such as CT and X-ray, to develop high-performance CAD systems.

Shahin et al.^14^ proposed a CAD system for detecting COVID-19 using CT images of patients, which grades the severity of the infection based on imaging data. The system comprises two main steps. In the first step, the authors used a modified version of the k-means algorithm to enhance the visibility and contrast of ground glass opacities. In the second step, they employed SVM to diagnose each patient. According to their experimental results, the system achieved an accuracy of 80%. On the other hand, Kogilavani et al.^15^ proposed a deep-learning CAD system that uses patients’ CT images for COVID-19 classification. They employed various convolutional neural network (CNN) architectures, including VGG16, DeseNet121, MobileNet, NASNet, Xception, and EfficientNet to classify patients into COVID-19 or non-COVID-19 groups. The authors used a dataset of 3833 patients collected from Kaggle to train and test their system. Their experimental results showed that the VGG16 architecture outperformed the other classifiers, achieving an accuracy of 97.68%. Yu et al.^16^ presented a COVID-19 severity identification system that utilized a patient’s CT volume to identify the severity of the disease. The system employed four deep-learning algorithms, including ResNet-50, Inception-V3, ResNet-101, and DenseNet-201, to extract features from CT images. These features were then fed into a machine learning algorithm to identify each case as severe or non-severe. The authors tested their system using a dataset consisting of 202 COVID-19-positive patients collected from three hospitals in Anhui, China. The authors evaluated their system using five machine learning algorithms, including linear SVM, KNN, linear discriminant, Adaboost decision tree, and cubic SVM. The results showed that using DenseNet-201 with a cubic SVM model outperformed the other models, achieving an accuracy of 95.20%. Nigam et al.^17^ proposed a deep-learning CAD system that could identify the presence of COVID-19 infection based on a patient’s X-ray. The system used VGG16, DenseNet121, Xception, NASNet, and EfficientNet architectures to detect COVID-19 infection. To evaluate the system’s performance, the authors used a dataset consisting of 16,634 patients collected from various hospitals in India. The results showed that the EfficientNet architecture outperformed the others, achieving an accuracy of 93.48%. Alqudah et al.^18^ conducted a study to compare the performance of several hybrid machine learning models, including CNNs, random forest (RF), SVM, and KNN, for the detection of COVID-19 from chest X-ray images. To evaluate the models’ performance, they used a dataset consisting of 1057 chest X-ray images, including 506 COVID-19-positive and 551 COVID-19-negative cases. The results showed that the CNN-based models outperformed the other models in terms of accuracy, sensitivity, specificity, and AUC. The best-performing CNN model achieved an accuracy of 96%, a sensitivity of 95%, a specificity of 97%, and an AUC of 0.99.

AI-based techniques have shown promise in assisting with the diagnosis and grading of COVID-19 severity using CT images, as demonstrated by the studies mentioned above. However, it is important to note that there are no published studies that specifically examine the correlation between radiology findings of COVID-19 lesions and the required respiratory support in infected patients. In this paper, we aim to investigate the potential correlation between the radiology features of COVID-19 lesions and the required respiratory support in infected patients. This investigation could provide valuable insights into the management of COVID-19 patients and may lead to improved treatment strategies. Below, will describe in detail the major steps of the proposed system.

## Method

**Figure 2:**
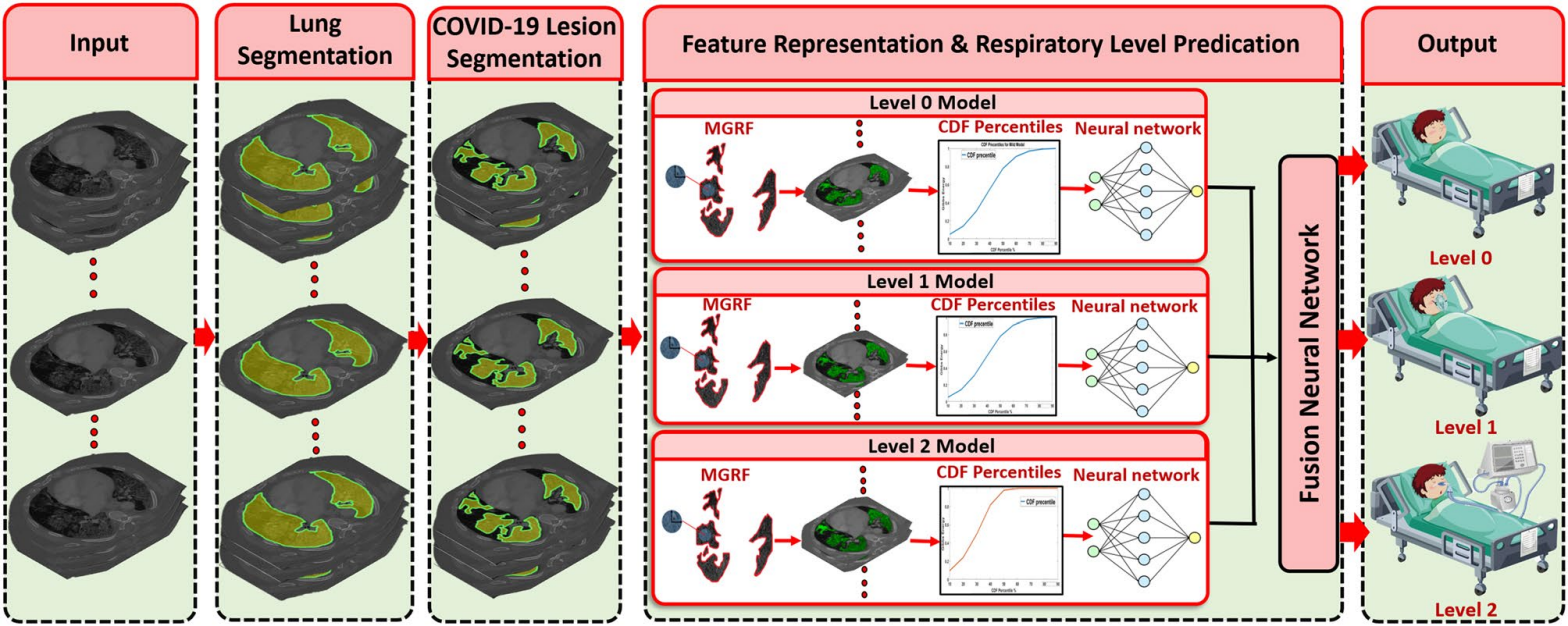
A schematic illustration of the proposed CAD system architecture.

In this paper, we proposed a new CAD system that predicts the respiratory support level required for each COVID-19 patient to recover from infection. The CAD system consists of four main steps. Firstly, the patient’s lung is segmented, followed by extraction of the lesion in the second step. To estimate the texture and morphology of the lesions, we trained an appearance model using the MGRF algorithm in the third step. The algorithm is applied three times to represent the lesion appearance at the three respiratory support levels. Finally, a two-stage neural network is used to diagnose and grade each patient into one of the three levels. Figure 2 illustrates the main steps of our CAD system.

### Lung segmentation

**Figure 3:**
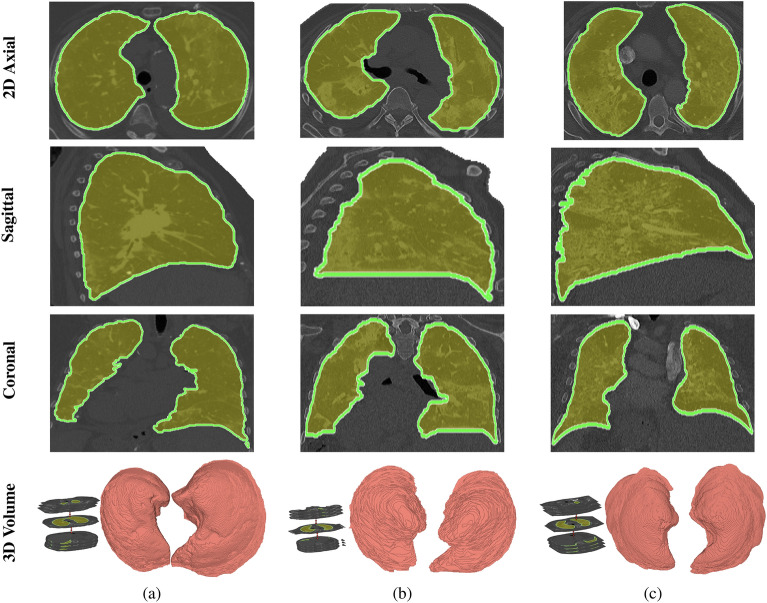
An illustrative example of the proposed segmentation approach for three patients requiring different levels of respiratory support during COVID-19 infection at 2D axial (first row), sagittal (second rows), and coronal (third row) cross-sections: (**a**) Level 0, (**b**) Level 1, and (**c**) Level 2. The images showcase the accuracy of the segmentation approach for various respiratory support levels.

To design a high-performance CAD system, we must use the markers extracted directly from COVID-lesions. To achieve this goal, we designed our system to start by extracting the lung region, followed by COVID-lesion segmentation. This two-step sequential design ensures accurate segmentation of COVID-lesions and avoids errors in cases where the lesions are close to the chest region. We achieved lung segmentation using our previously published method in^19^. This approach utilizes the first and second-order appearance models of lung and chest tissues to accurately segment the lung area, taking into account the similarity in appearance of certain lung tissues to other chest tissues such as bronchi and arteries. We extracted the first-order appearance model of the CT image using discrete Gaussian kernels and used a new version of the expectation-maximization algorithm to calculate the model parameters. The MGRF algorithm estimated the second-order appearance model by representing the pairwise interaction of the 3D lung tissues. Figure 3 shows the results of our segmentation method applied to three individuals with varying respiratory support levels needed during COVID-19 infection. For more details on our segmentation method, please refer to^19,20^.

### COVID-lesion segmentation

**Figure 4:**
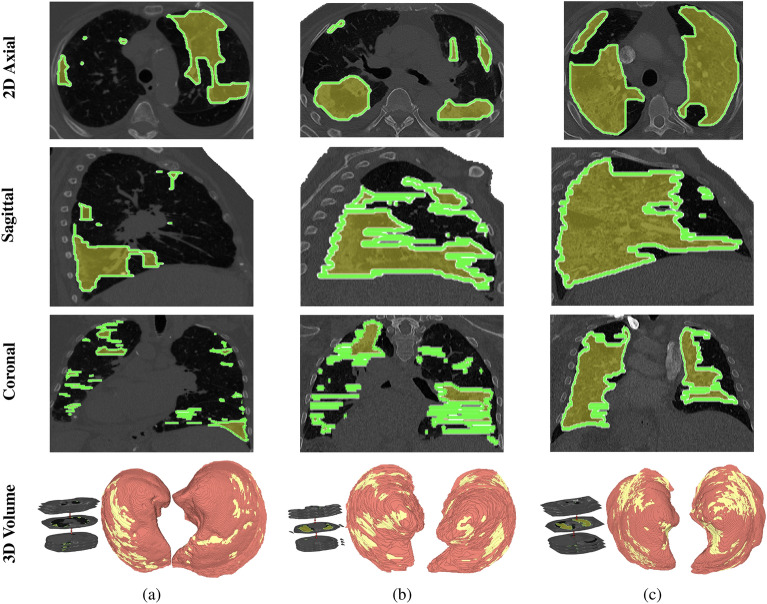
An illustrative example of the proposed lesion segmentation approach on three COVID-19 patients with varying respiratory support requirements at 2D axial (first row), sagittal (second rows), and coronal (third row) cross-sections: (**a**) minimal support (Level 0), (**b**) non-invasive support (Level 1), and (**c**) invasive support (Level 2).

First, an expert radiologist carefully selects the region of interest around the largest cross-section over COVID-19 lesions aiming to enhance the accuracy of lesion detection. Then, we used the segmentation models mentioned above to differentiate between normal lung tissues and COVID-19 lesions. In addition, we employed connected region analysis as an extra step. Figure 4 showcases some of the segmented COVID-19 lesions.

### Learning the appearance of COVID-19 lesions

**Figure 5:**
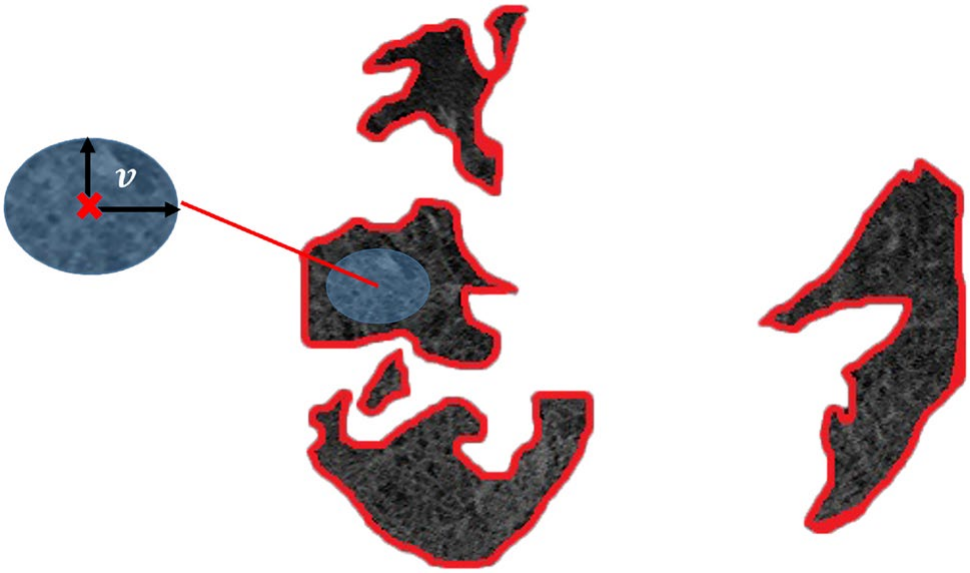
Central-symmetric neighborhood of a voxel within a CT slice. The neighborhood (orange band) of voxel *x* contains voxels within a distance $\nu \pm \frac{1}{2}$ of *x*.

The proposed approach considers each COVID-lesion as the realization of a piecewise stationary Markov–Gibbs random field (MGRF)^21–27^. The MGRF is constructed such that joint probabilities, i.e., voxel-voxel interactions, are central-symmetric. This means that the appearance of infected lung regions is modeled as a random process, in which the probability of each voxel being infected depends on the appearance of neighboring voxels. The use of a central-symmetric system of voxel–voxel interactions is crucial because it captures the circular symmetry of the lesion structure and allows for precise modeling of the appearance of COVID-19 lesions. Denote the neighborhood system of the MGRF by $\textbf{n}$; then every $n_{\nu } \in \textbf{n}, \nu = 1, \ldots , N$, is specified by a pair of positive real numbers $(\underline{d}_{\nu }, \overline{d}_{\nu })$. The $n_{\nu }$-neighborhood of voxel *x* is the set $\{x' \mid \underline{d}_{\nu } < \Vert x - x' \Vert \le \overline{d}_{\nu }\}$, where $\Vert \cdot \Vert$ denotes Euclidean distance. Figure 5 illustrates such a neighborhood system with $\underline{d}_{\nu } = \nu - \frac{1}{2}$ and $\overline{d}_{\nu } = \nu + \frac{1}{2}$.

Consider an image pair $(\textbf{g}, \textbf{m})$ from our training data set comprising a CT slice and its ground truth labeling, respectively. Denote by $\textbf{R}$ the set of “object” voxels, i.e. voxels within the infected lung region. Then the restricted neighborhood system is the set of voxels

$$\begin{aligned} c_{\nu } = \{(x,x') \mid x \in \textbf{R}, x' \in \textbf{R}, (x,x') \in n_{\nu }\} \end{aligned}$$

Finally let $f_{0}$ and $f_{\nu }$ denote empirical distributions (i.e., relative frequency histograms) of gray levels in $\textbf{R}$ and gray level co-occurrences (i.e., the frequency of co-occurrences) in $c_{\nu }$, respectively:^21,28^

1 $$\begin{aligned} f_{0}(q)= & {} \vert \textbf{R}\vert ^{-1} \left| \{x \in \textbf{R} \mid \textbf{g}(x) = q\} \right| ; \end{aligned}$$

2 $$\begin{aligned} f_{\nu }(q,q')= & {} \vert c_{\nu }\vert ^{-1} \left| \{(x,x') \in c_{\nu } \mid \textbf{g}(x) = q, \textbf{g}(x') = q'\} \right| . \end{aligned}$$

The empirical distribution (i.e., Eq. 1) represents the fundamental principle used to determine the frequency of each estimated Gibbs energy value within COVID lesions. Conversely, the frequency of co-occurrences (i.e., Eq. 2) signifies the joint occurrence frequency of two Gibbs energy values, which plays a pivotal role in constructing the second-order appearance of COVID-19 lesions.

The MGRF distribution of object voxel gray levels within an element of the training data $(\textbf{g}_{t}, \textbf{m}_{t}), t = 1, \ldots , T$ is the Gibbs distribution

3 $$\begin{aligned} \begin{aligned} P_t&= \frac{1}{Z_{t}} \exp \left( \sum \limits _{(x)\in \textbf{R}_t} \left( V_0\left( \textbf{g}_t(x)\right) + \sum \limits _{\nu =1}^{N} \sum \limits _{(x,x')\in c_{\nu ,t}} V_{\nu }\left( \textbf{g}_t(x),\textbf{g}_t(x')\right) \right) \right) \\&= \frac{1}{Z_{t}} \exp \left( \vert \textbf{R}_t\vert \left( \textbf{V}_{0,t}^\textsf{T}\textbf{F}_{0,t} + \sum \limits _{\nu =1}^{N}\rho _{\nu ,t}\textbf{V}_{\nu ,t}^\textsf{T} \textbf{F}_{\nu ,t} \right) \right) , \end{aligned} \end{aligned}$$

where $\rho _{\nu } = |c_{\nu }| / |\textbf{R}|$ is the mean size of the restricted neighborhoods relative to the size of the entire sublattice $\textbf{R}$. Since the premise of using the MGRF model is that lungs affected by a particular pathology (in this case COVID-19) will produce CT features alike in appearance, it is practical to approximate some of the quantities in Eq. (3) by their averages with respect to set of training images. Namely, $|\textbf{R}_{t}| \approx R_{\textsf{ob}} = \frac{1}{T}\sum _{t=1}^{T}|\textbf{R}_t|$, and $|c_{\nu ,t}| \approx c_{\nu ,\textsf{ob}} = \frac{1}{T}\sum _{t=1}^{T}|c_{\nu ,t}|$. With the condition that elements of the training set are statistically independent (e.g., if each CT image is taken from a different patient), the Gibbs distribution may be further simplified^28^:

$$\begin{aligned} P = \frac{1}{Z} \exp \left( TR_\textsf{ob} \left( \textbf{V}_{0}^\textsf{T} \textbf{F}_{0,\textsf{ob}}+ \sum \limits _{\nu =1}^{N}\rho _{\nu }\textbf{V}_{\nu }^\textsf{T} \textbf{F}_{\nu ,\textsf{ob}} \right) \right) . \end{aligned}$$

Here, the estimated restricted neighborhood size $\rho _{\nu } = c_{\nu ,\textsf{ob}} / R_\textsf{ob}$, and estimated weights $\textbf{F}_{0,\textsf{ob}}$ and $\textbf{F}_{\nu ,\textsf{ob}}$ again denote average values with respect to the set of training images.

Structural zeros can arise when there are little training data to identify the MGRF model, e.g., if only a relatively small lung region is affected by the disease. Then, by chance, some gray levels do not occur, or do not co-occur with certain other gray levels, in the training data and the corresponding elements of the weight vectors are zero. Test data where these values do occur then have zero likelihood. We deal with this potential problem by using pseudo-counts. For Eqs. (1) and (2), substitute the following, modified versions:

4 $$\begin{aligned} f_{0}(q)= & {} \frac{\left| \{(x)\in \textbf{R} \mid \textbf{g}(x) = q\} \right| + \varepsilon }{\vert \textbf{R}\vert + Q\varepsilon } \end{aligned}$$

5 $$\begin{aligned} f_{\nu }(q,q')= & {} \frac{\left| \{(x,x') \in c_{\nu } \mid \textbf{g}(x) = q, \textbf{g}(x') = q'\} \right| + \varepsilon }{\vert c_{\nu }\vert + Q^2\varepsilon }. \end{aligned}$$

Here *Q* is the number of discrete gray levels. The parameter $\varepsilon$ can be chosen according to several criteria. Following^28^, we set $\varepsilon$ to effect unit pseudo-count in the denominator, i.e. $\varepsilon := 1/Q$ (Eq. 4) or $\varepsilon := 1/Q^2$ (Eq. 5).

It remains only to estimate the Gibbs potentials. Using the same analytical approach as in^28^, these are approximated using the centered, training-set averaged, normalized histograms:

6 $$\begin{aligned} \begin{array}{llll} V_{0}(q) &{} = &{} \left( f_{0}(q)-\frac{1}{Q} \right) ; \\ V_{\nu }(q,q^{\prime }) &{} = &{} \left( f_{\nu }(q,q^{\prime })-\frac{1}{Q^{2}} \right) . \end{array} \end{aligned}$$

With the model now fully specified, the Gibbs energy of the lesion, or affected region of the lung, $\textbf{b}$ within a test image $\textbf{g}$ is obtained from the equation

7 $$\begin{aligned} E(\textbf{g},\textbf{b}) = \textbf{V}_{0}^\textsf{T} \textbf{F}_{0}(\textbf{g}, \textbf{b}) + \sum _{\nu \in \textbf{N}^\prime }\textbf{V}_{\nu }^\textsf{T} \textbf{F}_{\nu }(\textbf{g}, \textbf{b}). \end{aligned}$$

Here, $\textbf{N}^\prime$ is a selected top-rank index subset of the neighborhood system $\textbf{n}$, and $\textbf{F}_{0}(\textbf{g}, \textbf{b})$ and $\textbf{F}_{\nu }(\textbf{g}, \textbf{b})$ are just the histogram and normalized co-occurrence matrix, respectively, of object voxels in the test data. The training procedure for the MGRF model is summarized in Algorithm 1.

**Figure:**
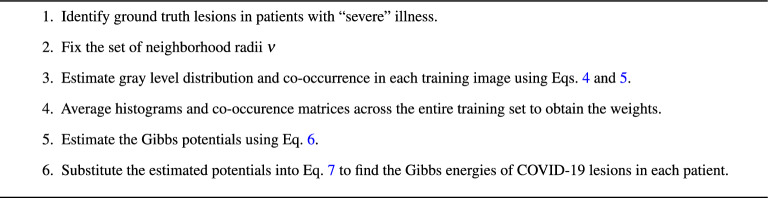
Algorithm 1. MGRF training model.

### Neural network based grading system

We designed a two-stage neural network to determine the level of respiratory support that each patient needed to recover from COVID-19. In the first stage, we use three feed-forward neural networks for training and testing the three estimated CDF percentiles separately. Therefore, we combined the testing results of the three neural networks and fed them into the final feed-forward neural network, in which we identified the appropriate respiratory support level needed for each patient. We used a backpropagation algorithm to train and test the four neural networks. Algorithm 2 shows the steps of the backpropagation method, which we followed to train our networks. Our neural network performance increased using the best values for the model’s hyperparameters. Several approaches can be used to identify the optimal values for hyperparameters. We used a random search strategy, which depended on randomly selected points from the hyperparameter space, and then tested the model’s performance on the training data for each random sample. The number of hidden layers in the feedforward network, the size of each hidden layer, and the hidden layer neurons’ activation function were all hyperparameters. A random search revealed that the rectified linear unit (ReLU) activation function and three hidden layers of 9, 10, and 21 neurons, respectively, were the values of the optimal hyperparameters for our system.

**Figure:**
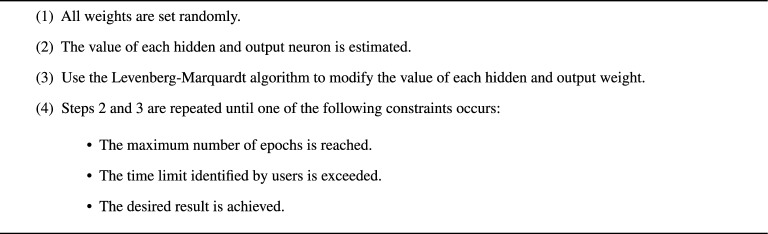
Algorithm 2. Backpropagation algorithm.

### Performance evaluation metrics

To evaluate the performance of our diagnostic system with three outcome levels, we used Cohen’s Kappa, which is a statistical measure of inter-rater agreement commonly used in such situations. One of the main benefits of using Cohen’s Kappa is its ability to take into account the possibility of chance agreement among raters, which is particularly important when the outcome prevalence is low or the categories are imbalanced. Moreover, Cohen’s Kappa can provide valuable insights into the sources of disagreement among raters, which can be used to improve the accuracy of the diagnostic system. The Cohen’s Kappa coefficient for a three-level diagnostic system is calculated as follows:

8 $$\begin{aligned} \text {Cohen's Kappa} = \frac{{Acc}_o - {Acc}_e}{1 - {Acc}_e} = 1 - \frac{1 - {Acc}_o}{1 - {Acc}_e} \end{aligned}$$

where ${Acc}_o$ is the observed accuracy between the output of the proposed diagnostic system and the ground truth and ${Acc}_e$ is the expected agreement between the outcome of the proposed diagnostic system and our gold standard reference standard. For a three-level diagnostic system, ${Acc}_o$ and ${Acc}_e$ are calculated as:

9 $$\begin{aligned} {Acc}_o = \frac{TP_{L2} + TP_{L1} + TP_{L0}}{N} \end{aligned}$$

and

10 $$\begin{aligned} \begin{aligned} {Acc}_e&= \left[ \frac{TP_{L2} + FN_{L2}}{N}\right] \\&\quad \times \left[ \frac{TP_{L2} + FP_{L2} + TP_{L1} + TP_{L0} + FN_{L1} + FN_{L0}}{N}\right] \\&\quad + \left[ \frac{TP_{L1} + FN_{L1}}{N}\right] \\&\quad \times \left[ \frac{TP_{L2} + TP_{L1} + TP_{L0} + FP_{L2} + FN_{L2} + FN_{L0}}{N}\right] \\&\quad + \left[ \frac{TP_{L0} + FN_{L0}}{N}\right] \\&\quad \times \left[ \frac{TP_{L2} + TP_{L1} + TP_{L0} + FP_{L2} + FN_{L2} + FN_{L1}}{N}\right] \end{aligned} \end{aligned}$$

where $TP_{L2}$ is the number of true positives for the Level 2 respiratory support, high-risk category, $TP_{L1}$ is the number of true positives for the Level 1 respiratory support, $TP_{L0}$ is the number of true positives for the Level 1 respiratory support, $FN_{L2}$ is the number of false negatives for the Level 2 respiratory support, $FN_{L1}$ is the number of false negatives for the Level 1 respiratory support, $FN_{L0}$ is the number of false negatives for the Level 0 respiratory support, $FP_{L2}$ is the number of false positives for the Level 2 respiratory support, and *N* is the total number of subjects.

In addition to reporting the observed accuracy and Cohen’s Kappa, we will report the true positive rate (i.e., sensitivity) and true negative rate (i.e., specificity) for each respiratory level.

All methods were carried out in accordance with relevant guidelines and regulations. The Institutional Review Board (IRB) of University of Louisville approved the study and its methods and confirmed that the research study followed all appropriate protocols and legal requirements. Patients (or, in the case of deceased patients, next of kin or legal guardian) provided informed consent.

## Experimental results

To test the performance of our proposed method, we collected a dataset consisting of 307 CT chest volumes from the University of Louisville, USA. Based on the support repository provided for each patient, the dataset was categorized into three distinct groups. These groups included 167 cases with minimum support (level 0), 69 cases with non-invasive support (level 1), and 71 cases with invasive support (level 2).

**Figure 6:**
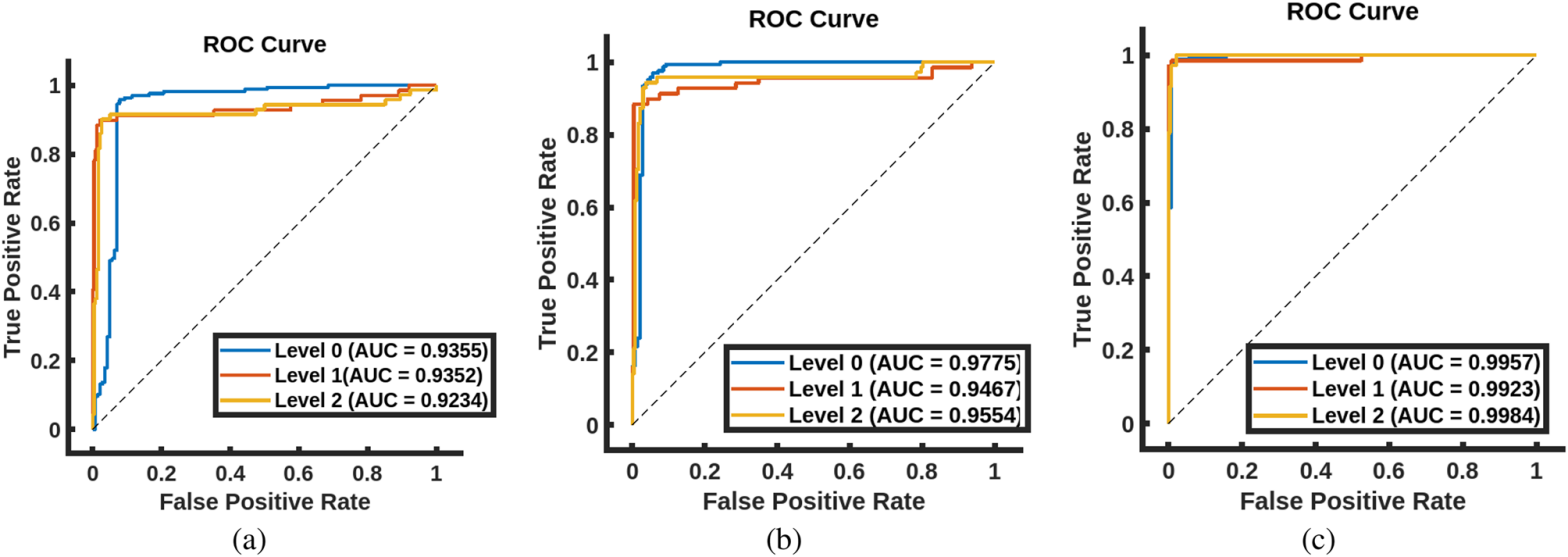
ROC curve of the proposed system using (**a**) 4-fold, (**b**) 5-fold, and (**c**) 10-fold cross-validation.

**Figure 7:**
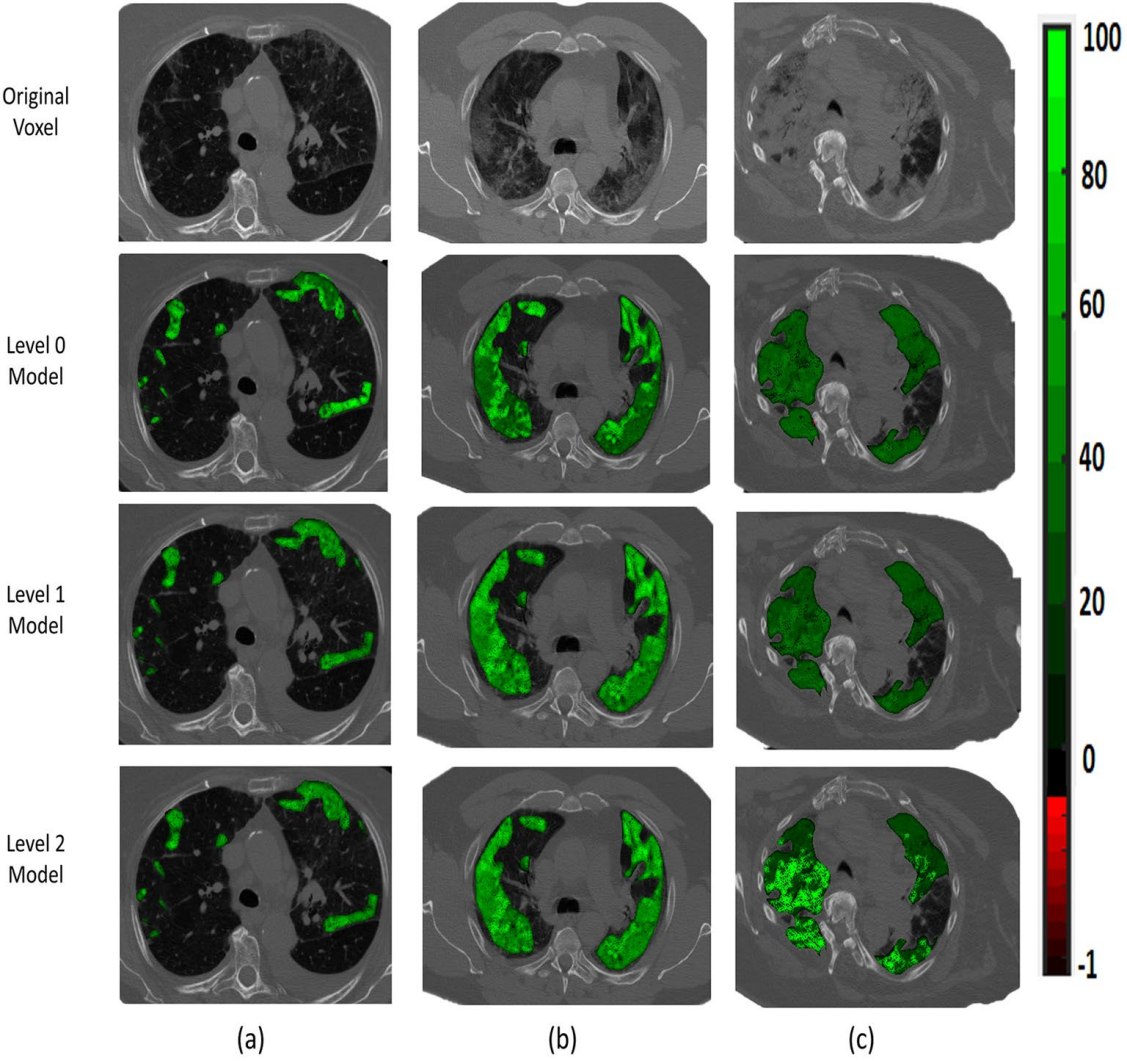
An illustrative color map example of Gibbs energies for (**a**) level 0, (**b**) level 1, or (**c**) level 2; tuned using level 0, level 1, or level 2 COVID-19 lesions.

To assess the performance of our CAD system, cross-validation approaches with 4, 5, and 10 folds are employed. Additionally, other machine learning classifiers are utilized to highlight the potential of our system performance. The performance of our system is shown in Table 1. As shown in Table 1, the proposed system outperform all other classifiers, achieving an accuracy of $97.72\pm 1.57$ and a kappa of $96.58 \pm 2.36$, using 10-fold cross-validation. Moreover, using 5-folds and 4-folds cross validation, the system consistently demonstrates a high accuracy of $93.79 \pm 1.37$ and $92.77 \pm 1.7$, and kappa of $90.68 \pm 2.05$ and $89.18 \pm 2.52$, respectively. These outcomes highlight the promise of the proposed system in predicting the respiratory support level for COVID-19 patients. Also, the area under the receiver operating characteristic (ROC) curve (AUC) for each of the three categories are estimated to show the performance of the proposed method in differentiating between the three categories, as shown in Fig. 6. The figure demonstrates the capability of the proposed method for distinguishing between the three levels, achieving AUC of 0.9957, 0.9923, and 0.9984 for levels 0, 1, and 2, respectively, using 10-fold cross-validation. To visually demonstrate the capability of the MRGF models in distinguishing between three levels, Fig. 7 shows the color map of Gibbs energy estimated from three MGRF models for three patients with different respiratory support requirements. As shown in the figure, the Gibbs energy estimated from the MGRF model tuned using a given level of respiratory support is higher for a patient requiring that particular level, compared to other models. This confirms the capability of the proposed system in effectively differentiating between the three levels of respiratory support.

**Table 1:** Comparison between the proposed system and different machine learning techniques.

	Classifier	Metrics	Class evaluation			Overall evaluation	
			Level 0	Level 1	Level 2	Overall accuracy (%)	Kohen Kappa (%)
4-fold	Random forest^29^	Sensitivity (%)	$90.79 \pm 8.89$	$57.80 \pm 6.24$	$62.39 \pm 10.82$	$76.81 \pm 3.84$	$61.04 \pm 6.06$
		Specificity (%)	$71.74 \pm 8.13$	$95.18 \pm 3.77$	$91.43 \pm 5.4$		
	Decision trees^30^	Sensitivity (%)	$83.27 \pm 7.98$	$51.46 \pm 9.29$	$46.04 \pm 17.58$	$67.53 \pm 7.04$	$45.88 \pm 11.25$
		Specificity (%)	$70.8 \pm 6.19$	$86.55 \pm 7.74$	$88.63 \pm 5.83$		
	Naive Bayes^31^	Sensitivity (%)	$88.29 \pm 5.07$	$67.28 \pm 7.57$	$65.17 \pm 13.4$	$78.23 \pm 4.53$	$63.98 \pm 7.29$
		Specificity (%)	$78.49 \pm 13.14$	$93.89 \pm 2.19$	$90.52 \pm 3.38$		
	SVM^32^	Sensitivity (%)	$90.11 \pm 5.6$	$63.29 \pm 5.72$	$67.87 \pm 14.51$	$78.94 \pm 1.4$	$64.77 \pm 1.62$
		Specificity (%)	$76.67 \pm 8.09$	$95.14 \pm 2.46$	$91.32 \pm 2.22$		
	KNN^33^	Sensitivity (%)	$90.79 \pm 7.00$	$68.23 \pm 17.28$	$67.97 \pm 17.59$	$78.91 \pm 1.89$	$64.5 \pm 3.08$
		Specificity (%)	$72.4 \pm 9.31$	$95.54 \pm 2.28$	$93.42 \pm 3.89$		
	AdaBoost^34^	Sensitivity (%)	$91.36 \pm 7.95$	$62.07 \pm 8.79$	$49.93 \pm 15.53$	$75.18 \pm 4.21$	$58.62 \pm 7.13$
		Specificity (%)	$71.12 \pm 9.5$	$92.26 \pm 2.84$	$92.65 \pm 4.15$		
	Proposed system	Sensitivity (%)	${\textbf {93.27}} \pm {\textbf {3.68}}$	${\textbf {92}} \pm {\textbf {0}}$	${\textbf {93}} \pm {\textbf {2}}$	${\textbf {92.77}} \pm {\textbf {1.7}}$	${\textbf {89.18}} \pm {\textbf {2.52}}$
		Specificity (%)	${\textbf {95}} \pm {\textbf {1.15}}$	${\textbf {97.55}} \pm {\textbf {1.88}}$	${\textbf {96.57}} \pm {\textbf {0.98}}$		
5-fold	Random forest^29^	Sensitivity (%)	$92.47 \pm 6.67$	$63.77 \pm 11.53$	$64.23 \pm 13.48$	$79.46 \pm 2.36$	$65.60 \pm 4.62$
		Specificity (%)	$73.29 \pm 10.11$	$95.95 \pm 2.78$	$93.17 \pm 5.38$		
	Decision trees^30^	Sensitivity (%)	$78.93 \pm 8.95$	$60.27 \pm 13.82$	$43.90 \pm 17.26$	$66.61 \pm 5.13$	$45.90 \pm 9.16$
		Specificity (%)	$71.43 \pm 9.86$	$86.14 \pm 5.89$	$87.41 \pm 9.39$		
	Naive Bayes^31^	Sensitivity (%)	$89.40 \pm 3.95$	$66.20 \pm 10.97$	$62.98 \pm 15.64$	$78.08 \pm 3.56$	$63.57 \pm 5.87$
		Specificity (%)	$77.96 \pm 13.38$	$93.48 \pm 3.21$	$91.00 \pm 3.77$		
	SVM^32^	Sensitivity (%)	$92.40 \pm 3.29$	$69.36 \pm 11.85$	$63.27 \pm 9.88$	$80.43 \pm 2.17$	$67.37 \pm 4.33$
		Specificity (%)	$77.5 \pm 8.55$	$95.18 \pm 3.03$	$92.68 \pm 3.26$		
	KNN^33^	Sensitivity (%)	$94.72 \pm 2.43$	$63.77 \pm 11.53$	$61.73 \pm 12.07$	$80.03 \pm 1.54$	$66.52 \pm 3.04$
		Specificity (%)	$71.33 \pm 9.41$	$96.34 \pm 0.80$	$94.71 \pm 2.95$		
	AdaBoost^34^	Sensitivity (%)	$91.88 \pm 5.56$	$62.11 \pm 11.61$	$49.81 \pm 15.62$	$75.37 \pm 3.51$	$59.12 \pm 5.85$
		Specificity (%)	$70.01 \pm 14.42$	$92.72 \pm 3.27$	$92.43 \pm 6.25$		
	Proposed system	Sensitivity (%)	${\textbf {92.38}} \pm {\textbf {2.61}}$	${\textbf {93.05}} \pm {\textbf {2.78}}$	${\textbf {96}} \pm {\textbf {2.24}}$	${\textbf {93.79}} \pm {\textbf {1.37}}$	${\textbf {90.68}} \pm {\textbf {2.05}}$
		Specificity (%)	${\textbf {96.52}} \pm {\textbf {1.34}}$	${\textbf {97.56}} \pm {\textbf {1.73}}$	${\textbf {96.6}} \pm {\textbf {2.19}}$		
10-fold	Random forest^29^	Sensitivity (%)	$91.54 \pm 18.57$	$67.03 \pm 7.02$	$65.39 \pm 16.86$	$79.95 \pm 4.94$	$66.5 \pm 9.01$
		Specificity (%)	$77.84 \pm 6.51$	$94.91 \pm 14.61$	$92.16 \pm 3.77$		
	Decision trees^30^	Sensitivity (%)	$83.55 \pm 19.80$	$61.42 \pm 9.31$	$51.98 \pm 22.75$	$71.08 \pm 6.97$	$52.75 \pm 12.43$
		Specificity (%)	$71.12 \pm 6.86$	$91.14 \pm 15.09$	$88.53 \pm 6.30$		
	Naive Bayes^31^	Sensitivity (%)	$90.28 \pm 21.38$	$68.46 \pm 6.91$	$63.69 \pm 14.36$	$79.26 \pm 3.65$	$65.62 \pm 6.94$
		Specificity (%)	$78.51 \pm 5.56$	$93.74 \pm 14.52$	$92.07 \pm 5.73$		
	SVM^32^	Sensitivity (%)	$92.54 \pm 21.83$	$67.71 \pm 5.73$	$65.25 \pm 18.28$	$80.63 \pm 5.77$	$67.57 \pm 11.09$
		Specificity (%)	$76.75 \pm 4.62$	$95.83 \pm 17.16$	$92.73 \pm 3.72$		
	KNN^33^	Sensitivity (%)	$94.90 \pm 24.36$	$64.67 \pm 4.46$	$65.43 \pm 10.29$	$81.14 \pm 4.82$	$68.45 \pm 8.41$
		Specificity (%)	$73.56 \pm 4.69$	$96.81 \pm 11.73$	$94.52 \pm 2.37$		
	AdaBoost^34^	Sensitivity (%)	$86.04 \pm 21.96$	$64.53 \pm 19.02$	$60.69 \pm 18.44$	$77.13 \pm 5.54$	$61.89 \pm 10.16$
		Specificity (%)	$71.52 \pm 3.74$	$92.2 \pm 17.29$	$94.94 \pm 4.14$		
	Proposed system	Sensitivity (%)	${\textbf {96.27}} \pm {\textbf {4.82}}$	${\textbf {99}} \pm {\textbf {3.16}}$	${\textbf {98}} \pm {\textbf {4.26}}$	${\textbf {97.72}} \pm {\textbf {1.57}}$	${\textbf {96.58}} \pm {\textbf {2.36}}$
		Specificity (%)	${\textbf {99.5}} \pm {\textbf {1.58}}$	${\textbf {98.55}} \pm {\textbf {2.34}}$	${\textbf {98.55}} \pm {\textbf {2.34}}$		

Significant values are in [bold].

**Figure 8:**
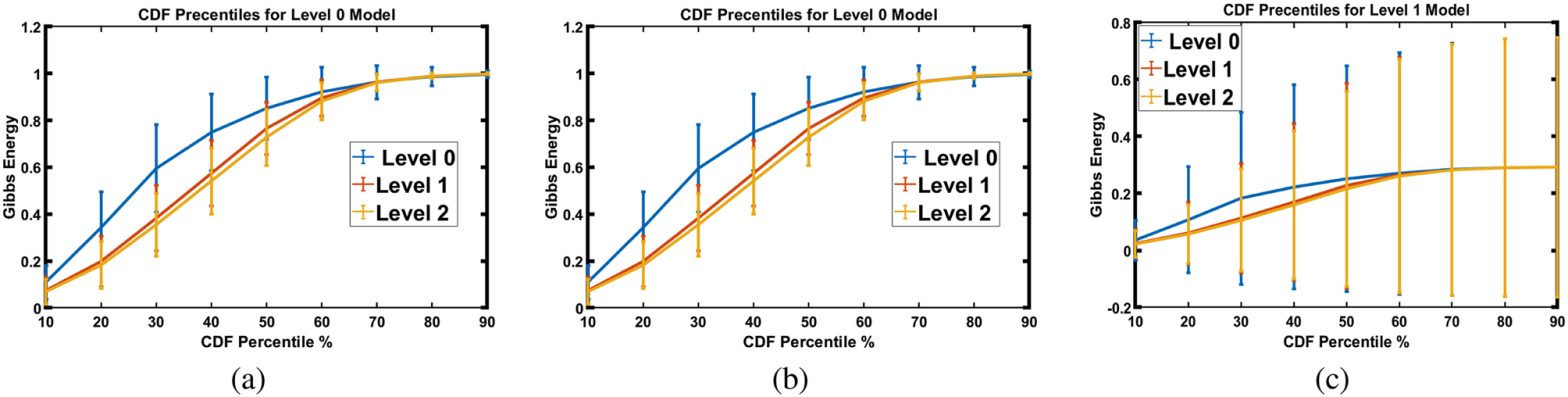
Estimated error average of CDF percentiles for three levels when tuning MGRF parameters using (**a**) level 0, (**b**) level 1, or (**c**) level 2.

**Figure 9:**
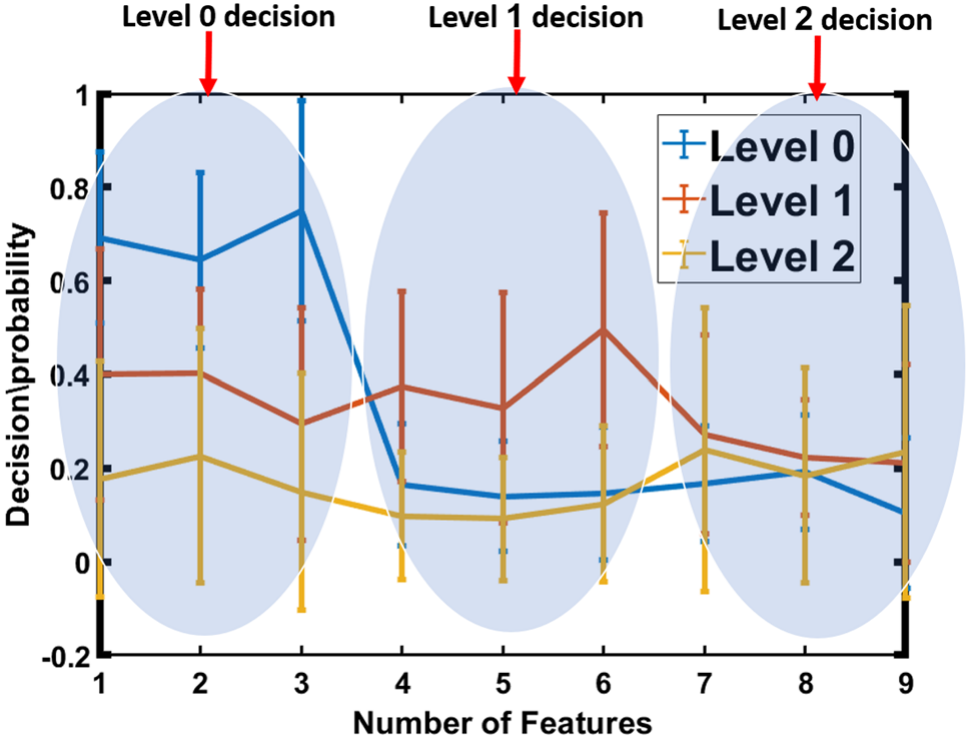
Estimated error average of traing data that represent each patient.

Due to the varying length of Gibbs energy for each patient is different, CDF percentiles are utilized as new, scale-invariant representations of the estimated Gibbs energy. Figure 8 shows the mean and standard deviation of CDF percentile for three respiratory support levels, as estimated from three models. As shown in the figure, representing the patient with the CDF percentiles leads to an obvious separation between the three respiratory support levels, which demonstrates the system’s ability to effectively distinguish between the different COVID-19 respiratory support levels. However, some of these features partially overlap with those associated with different respiratory support levels. Thus, the decisions/probabilities of each model are fused using another neural network to increase the system’s performance and separability between the three levels, as presented in Fig. 9. As demonstrated in the figure, it is easy for the fused neural network to predict the respiratory support level needed for each patient. All of these results show the system’s accuracy and effectiveness in correctly predicting the patient’s need for respiratory support based on CT scans. Hence, this could result in more precise diagnoses, better treatment strategies, and better patient outcomes.

## Discussion

Individuals with COVID-19 infection suffer from respiratory impairments and Acute Respiratory Distress Syndrome (ARDS). So, many COVID-19 patients need mechanical ventilation or soft oxygen to recover from the infection^35^. COVID-19 patients who need an intensive care unit (ICU) may suffer from acute renal failure and organ dysfunction, such as heart failure. Unfortunately, the death rate of COVID-19 infection for patients who needed ICU reached 97% at the early stages of the epidemic^36,37^. So, identifying the individuals that need ICU from the beginning of the COVID-19 infection is the only way to fight COVID-19 and reduce its death rate. This paper proposes a new, fast (with an average processing time of $66.55 \pm 25.06$ s), automated, and accurate CAD system that predicts the respiratory support level needed for each patient to recover from COVID-19 infection. To do this, this system identifies the correlation between the patient’s CT image and the respiratory support level. Our empirical results show that our proposed method achieved an accuracy of 97.72%. Several related studies used AI techniques to diagnose or grade the severity of COVID-19 infection using X-rays, CT scans, or medical data^9–18,38–43^, or to diagnose other diseases^44–54^. In these studies, many machine learning algorithms are adopted, such as CNN, SVM, KNN, VGG16, and Xception. These studies attained accuracy rates of 92.0–97.68%. However, they suffer from some drawbacks: (1) many recent works are based on deep learning algorithms with many convolution layers to perform feature extraction steps, which may increase the time required to identify COVID-19 severity. The increase in processing time is an acceptable trade-off, since deep learning automatically extracts relevant features instead of relying on a prior set of hand-crafted features. (2) Several existing studies have proposed a CAD system to classify the COVID-19 infection without determining the disease severity. (3) All recent works were based on clinical or imaging data without showing the correlation between these data and the repository support level that each patient had already preserved. AI has demonstrated its value in medical applications and gained widespread acceptance due to its high accuracy and predictability. By synergistically incorporating AI with chest imaging and other clinical data, it has the potential to significantly augment therapeutic outcomes. AI holds significant potential in detecting lung inflammation in CT medical imaging during the COVID-19 diagnosis stage. Furthermore, it offers the capability to precisely segment areas of interest from CT scans, further advancing the diagnostic process. Thus, acquiring self-learned characteristics for diagnosis or other applications becomes straightforward. By integrating multimodal data, including clinical and epidemiological information, within an AI framework, it becomes feasible to generate comprehensive insights for detecting and treating COVID-19 patients and potentially curbing the spread of this pandemic.

## Conclusions

As mentioned in this paper, it is evident that COVID-19 resulted in a significant mortality rate primarily attributed to the erroneous or delayed determination of the necessary level of respiratory support for each patient. So, utilizing AI techniques to accurately identify the necessary respiratory support level for each patient at the onset of the infection becomes an imperative and unavoidable. In this paper, we proposed a new CAD system that utilizes CT chest volumes to accurately predict the required respiratory support level for each COVID-19 patient’s recovery. Once the respiratory support level is determined for each patient, physicians can then provide personalized treatment recommendations, which have the potential to significantly reduce mortality rates. The results of this study demonstrated the promise of integrating spatial MGRF model with machine learning to predict respiratory support level in COVID-19 patients. However, this study has some limitations. One of them is the need for improvement in lesion segmentation, as the proposed segmentation relies heavily on the selection of the region of interest by a radiologist. Furthermore, additional investigation is necessary to assess how well the system performs with an external dataset. In the future, we intend to propose a fully automatic lesion segmentation system as well as improve the proposed system’s accuracy by extracting additional features that can be discovered through deep learning. Moreover, we intend to assess the efficacy of the grading system used in this paper (i.e., the levels of respiratory support) by conducting a comparative analysis with established radiology grading techniques.

## Data Availability

Correspondence and requests for materials should be addressed to Ayman El-Baz.
